# Salivary glucose as a non-invasive biomarker of type 2 diabetes mellitus

**DOI:** 10.4317/jced.55009

**Published:** 2018-09-01

**Authors:** Raphael-Enrique Tiongco, Aira Bituin, Engracia Arceo, Nicole Rivera, Eloisa Singian

**Affiliations:** 1 Department of Medical Technology, College of Allied Medical Professions, Angeles University Foundation, Angeles City, 2009, Philippines

## Abstract

**Background:**

Every year, millions of people are diagnosed with Diabetes mellitus (DM) and the number of new and undiagnosed cases continue to rise. Diagnosis of diabetes is usually performed by blood glucose analysis after fasting for a certain period. However, this method uses an invasive technique that can cause discomfort and even trauma to some patients which could eventually lead to behavioral changes such as avoidance of healthcare and laboratory testing. Studies that explore the diagnostic value of salivary glucose are promising due to the non-invasiveness of the test procedures and its potential correlation with blood results.

**Material and Methods:**

The study conducted aimed to determine if salivary glucose can be utilized as an alternative to blood glucose in the screening, diagnosis, and monitoring of type 2 diabetes mellitus (T2DM). A total of 75 participants were recruited and equally divided into 3 groups (normal fasting glucose, impaired fasting glucose, and provisional DM) based on their fasting blood glucose (FBG) level. Blood and unstimulated saliva were collected from each participant and were subjected to glucose analysis using the routine glucose oxidase-peroxidase method.

**Results:**

Using Pearson’s correlation and linear regression, a high degree and significant correlation was observed between blood and salivary glucose (r = 0.715, *p*<0.001). Further analysis showed that salivary glucose is 88.5% sensitive and 61.5% specific with a positive predictive value of 45.8%, and a negative predictive value of 97.1%.

**Conclusions:**

Salivary glucose is comparable to blood glucose in diagnosing and monitoring T2DM and is considered more advantageous than blood due to its non-invasive nature.

** Key words:**Saliva, glucose, non-invasive, diabetes.

## Introduction

Diabetes remains to be a global health concern with an estimated of 422 million people affected worldwide (1). In the Philippines, while there are more than 4 million Filipino adults reported with the disease, a large unknown number remains undiagnosed. The figure translates to one in every five Filipinos likely to have diabetes or pre-diabetes (2). Early and effective screening of diabetes is an important strategy to reduce the incidence of the disease and its complications. Blood testing remains to be the gold standard in the diagnosis but this can be invasive and painful for most patients leading to anxiety, risk of infection and the need for skilled phlebotomist (3). This painful experience could lead to profound health, societal, psychological, and social consequences and is highly associated with an avoidance behavior. Fear and anxiety to needles may result in non-compliance with health care services, such as performance of blood tests (4).

Studies that explore the diagnostic value of salivary glucose are promising due to the non-invasiveness of the test procedure and its potential correlation with blood results. The human saliva, an exocrine fluid secretion, has high potential for screening health and diseases (5,6). It consists of water, electrolytes and variety of proteins like enzymes, immunoglobulins, albumin, some polypeptides and biomarkers which can be useful for rapid tests (5,6). Studies show that proteins present in blood are present in saliva as well (5). Therefore, saliva is functionally comparable to blood in reflecting the physiological status of the body (7). Because of its potential clinical value, there is an increase use of saliva in the diagnosis for diseases (6). The collection of saliva is also easier and non-invasive compared to collection of blood (5,7).

The study was conducted to determine if salivary glucose can be used as a marker for screening, diagnosing, and monitoring type 2 diabetes mellitus (T2DM). Specifically, it aimed to establish a correlation between variables by determining the variation of salivary glucose based on changes to the participant’s blood glucose levels. The current study was conducted in order to assess the potential role of saliva as a diagnostic tool by correlating the levels of blood and salivary glucose among individuals with normal fasting glucose (NFG), impaired fasting glucose (IFG), and provisional DM (PDM).

## Material and Methods

-Research Design

A cross-sectional study design was used to compare the levels of salivary glucose among individuals with NFG, IFG, and PDM. The possible association of salivary glucose with blood glucose was also identified.

-Research Locale

The study was conducted at the Department of Medical Technology of Angeles University Foundation. Participants of the study included 25 males and 50 non-pregnant females with ages ranging from 31 to 61 years old and are currently residing in Angeles City, Philippines.

-Research Respondents 

A total of 86 participants were initially recruited by the researchers and were divided into three groups based on their FBG level. However, to equalize the number of participants across all the groups, 11 were randomly excluded (n = 75). Post-hoc power analysis using G*Power version 3.0.10 was performed and showed that based on the number of respondents acquired, the statistical power of the study is 1.00 at two-sided 0.05 level of significance with an effect size of 1.00.

Participants included in the study are males and non-pregnant females ages 31 years old and above and were willing to participate in the study. Subjects excluded are those with: Type 1 DM, renal disease undergoing hemodialysis, diagnosed with endocrine disorders, cancer, cardiovascular disease, communicable disease, and those undergoing insulin therapy (8,9). All participants who passed the inclusion criteria were grouped based on their FBG level. FBG-based grouping scheme is based on the latest clinical practice guidelines of the American Diabetes Association. Respondents with an FBG level of <100 mg/dL were classified under Group 1 or those with a normal fasting glucose (NFG), those with an FBG between 100 to 125 mg/dL were classified under Group 2 or those with an impaired fasting glucose (IFG), and those with an FBG of greater than or equal to 126 mg/dL were classified under Group 3 or those who have provisional diabetes mellitus (PDM) (9).

-Ethical Approval

All procedures performed in this study were in accordance with the ethical standards of the Angeles University Foundation – Center for Research and Development Ethics Review Committee (AUF-CRD ERC). Informed consent was obtained from all individual participants included in the study.

-Patient Preparation and Sample Collection 

Prior to specimen collection, participants were asked to fast for a period of 6 to 8 hours. Fasting is essential to ensure that all analytes tested are at their basal state. Before collection of saliva, participants were asked to rinse their mouth with distilled water 2 times. Whole saliva was collected by passive drooling. Participants were instructed to spit the pooled saliva in a sterile, disposable plastic container over a period of 5 minutes. Samples were then stored on ice and was sent to the laboratory immediately. Samples were centrifuged at 2000 RPM for about 5 minutes. Fasting blood samples were also drawn from all participants under aseptic condition and both samples were then immediately tested for the level of glucose (10-12).

-Glucose Estimation

The method used for the estimation of glucose in blood and saliva were based on the studies of Gupta *et al.* (13), Mascarenhas *et al.* (14), Soares *et al.* (15), and Ladgotra *et al.* (11) which utilized routine glucose oxidase-peroxidase end-point method. Concentration of the analyte was calculated based on the optical density readings obtained after spectroscopic analysis.

-Statistical Analysis

SPSS version 20 was used to analyzed the acquired data. Pearson’s correlation and linear regression was used to determine the relationship of salivary glucose with blood glucose. One-way analysis of variance (ANOVA) with Scheffe’s Test was used to compare the means of both demographic and clinical characteristics across the three groups. MedCalc was used to plot for the receiver operating characteristic (ROC) curve. A cut-off point with the highest sensitivity, specificity, positive and negative predictive value in diagnosing T2DM was also determined.

## Results

**Figure 1:**
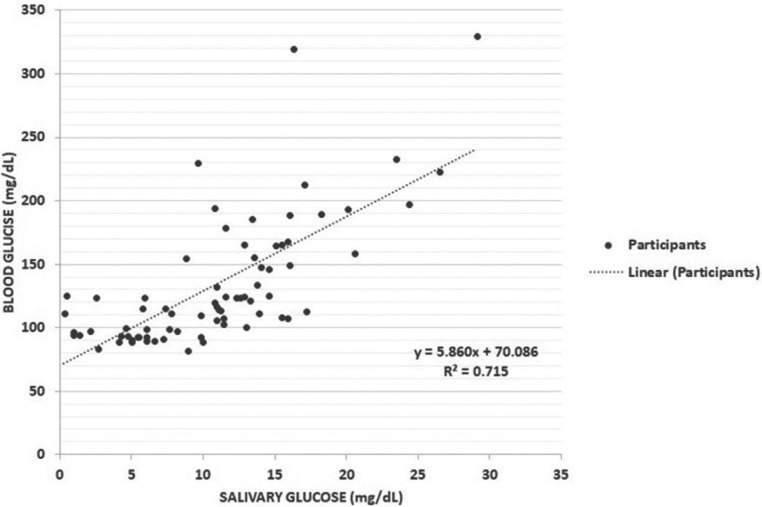
Scatter plot demonstrating the correlation between FBG and salivary glucose.

Figure 1 summarizes and illustrates the correlational analysis using Pearson’s Coefficient between FBG and salivary glucose. Correlation coefficient between salivary and blood glucose was computed with significant r value of 0.715 (*p* < 0.001).

Linear regression analysis was also performed and showed that salivary glucose (*p* < 0.001) can significantly predict the value of blood glucose. Hence, from a given value of salivary glucose, one can compute the blood glucose level by using the equation obtained from the linear regression analysis. The formula is: [Blood Glucose (mg/dL) = 70.086 + (5.860 x Salivary Glucose)].

One-way analysis of variance of age, blood, and salivary glucose among the study group was performed ( Table 1). As shown below, there was no significant difference for age (*p* = 0.125) across all three groups. However, significant differences were observed with the means of both blood (*p* < 0.001) and salivary glucose (*p* < 0.001).

**Table 1:**
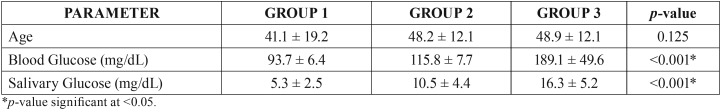
One-way ANOVA testing of age, blood and salivary glucose among the study group.

Post-hoc testing was also performed in order to determine the exact degree of difference among the study group ( Table 2). The mean salivary and blood glucose levels of group 2 (IFG) is significantly lower compared to group 3 (PDM), but is significantly higher when compared to group 1 (NFG) (1 < 2 < 3). This suggests that blood glucose correlates with the levels of glucose in the saliva.

**Table 2:**
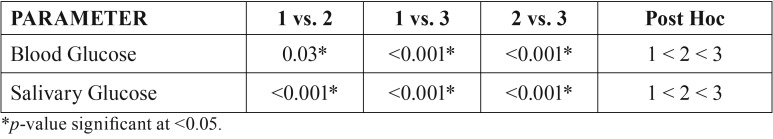
Post-ANOVA testing using Scheffe’s test.

**Figure 2:**
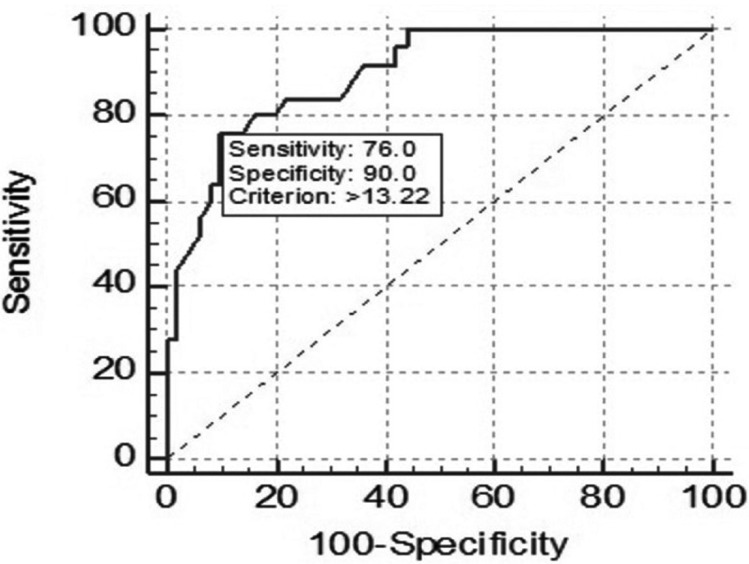
ROC curve analysis of salivary glucose.

Cut-off value to diagnose type 2 diabetes mellitus using salivary glucose was determined using the ROC curve is shown in Figure 2. Among the given cut-off points, the optimal value of salivary glucose was >13.22 mg/dL which may translate to the idea that patients with salivary glucose above this value are most likely to be diabetic.

The ROC curve also yielded a sensitivity of 76.0%, specificity of 90.0%, positive predictive value of 45.8%, and a negative predictive value of 97.1%. These values will tell that salivary glucose would correctly identify 45.8% of the population with high plasma glucose levels. Conversely, the false-negative rate was small relative to the true negative rate, so the probability of an individual having a high plasma glucose level with a low salivary glucose level is very low, and the negative predictive value is high (97.1%).

## Discussion

Salivary glucose has been investigated by various studies as a non-invasive alternative in overcoming the problems associated with blood glucose monitoring. But because contradicting results are obtained, the idea of saliva being an effective replacement for blood glucose is still debatable (16-20). Several studies suggest that significant correlation exist between salivary and blood glucose and would be helpful in monitoring diabetes. Considering that the procedures for glucose monitoring today are invasive, saliva can therefore serve as an alternative non-invasive diagnostic fluid to help overcome this problem. Saliva testing therefore surpasses all of the limitations of venipuncture and provides the ease of testing among all age groups (21-23). However, even with the existing data regarding the advantage of saliva testing and the significant correlation of blood and salivary analytes, the current findings still contradict with previous studies. A study conducted by Englander *et al.* (24) expressed their doubt about replacing blood glucose with parotid saliva secretion in the diagnosis of diabetes mellitus due to its low concentration. Another study conducted by Lopez *et al.* (25) showed that no significant correlation were found between salivary glucose level and HbA1c percentage.

In the study, researchers were able to establish a positive correlation between FBG and salivary glucose (r = 0.715, *p* < 0.001). Hence, due to the significant positive correlation seen between salivary glucose and FBG, using the equation derived in the linear regression analysis, blood glucose can be predicted using a formula with only the salivary glucose levels as the available data. The results are similar with previous studies which confirms higher levels of salivary glucose in diabetic patients compared with healthy controls (21-23). Quoting the works of several researchers, Abikshyeet *et al.* (21) summarized that there is no single mechanism to explain the appearance of glucose in the saliva during periods of prolonged hyperglycemia. The small molecular size, possible damage in the permeability of basement membrane, changes in the blood vessels, increased leakage from the ductal cells and leakage through the gingival crevices may all contribute to the multifactorial cause of increased levels of salivary glucose.

Laboratory analyses using saliva as the specimen surpasses all of the limitations of performing venipuncture and provides the ease of testing among all age groups (14). Tests done on saliva have already made significant exposure in the field of diagnostics. Testing of antibodies, unconjugated steroids, hormones and certain drugs are some of the adequate evidence that saliva reflects blood concentrations of substances accurately. Considering that blood is the standard for screening, monitoring and diagnosing diabetes, saliva, however, transcends several of its disadvantages and fulfils diagnostic concerns as it closely reflects blood levels (26,27).

As noted in the PPV and NPV, saliva can certainly identify the population whose blood glucose levels are within the normal range, thus, sparing them from further invasive testing. This strategy will negate the previous statements that saliva cannot be effectively used as a screening tool for diabetes. Instead, the specimen can be used as an initial screening for healthy individuals and also a monitoring tool for patients with known hyperglycemia. The findings of the study is similar with the conclusion made by Hartman *et al.* (10) that saliva can be used as a screening assay for high fasting glucose level. However, the presence of co-morbidity, compliance to treatment, and the small number of samples may contribute to the possibility of type II statistical error that cannot be ruled out which calls for a more extensive sample to substantiate the usefulness of salivary glucose as a biomarker for T2DM.

## Conclusions

Saliva is comparable to blood in terms of screening and diagnosing T2DM. Thus, salivary glucose may be used as a non-invasive biomarker for screening, diagnosing and monitoring T2DM. Since collection of saliva samples is safe and easy, further studies should be conducted to assess the potential of saliva as an alternative diagnostic fluid. To the best of our knowledge, there are no studies available in peer-reviewed journals regarding salivary glucose and its relation to T2DM conducted in the Philippines. Furthermore, the researchers recommend further investigations that would fully support the claims of this study. A longitudinal large-scale study should be conducted due to the limitations of this cross-sectional study. Other parameters such as glycemic control, insulin resistance, and metabolic syndrome are also recommended to be included. A more diverse population among Filipinos and a homogenous age group are also suggested. The researchers also recommend future studies to elaborate further other salivary analytes that can be used for the diagnosis of T2DM as well as other factors that contribute to their excretion in saliva.
